# Treatment of Simple Acne

**Published:** 1899-03

**Authors:** 


### Treatment of Simple Acne.

McKinney (*Maryland Med. Jour.*, November 19, 1898) advocates at least three hot baths each week taken at night followed by cold sponging, and a cold sponge bath every morning for patients who are troubled with acne. A light nutritious diet, plenty of exercise, and bowels kept regular, with cascara if necessary, are points not to be forgotten in any case. Tonics, cod-liver oil, phosphates, etc., may be required by the particular patient. The remedy par excellence for acne simplex is calcium sulphid. The proper dose is one-half of a grain, twice daily, and this dose should be steadily increased until four such tablets are taken each day. If the taste is objected to, it may be disguised by sugar coating, or the drug may be given in capsules. In case of excessive gastric irritation, it may be desirable to begin treatment with one-tenth or one-fifth of a grain. In the acute stages of the trouble, arsenic does no good, and may do actual harm. At each visit it is well for the physician to spend a little time in gently squeezing out the larger comedones, and curetting the smaller ones with the comedone extractor. The pustules should be lanced at the base in a slanting direction, and the point of the needle or lancet, swung around in the abscess cav-

*A Treatise on Fractures and Dislocations. Lewis A. Stimson, M. A., M. D., Professor of Surgery in Cornell University Medical College. Lea Brothers & Co. Just Issued.

be squeezed out without disturbing the overlaying crust, the resultant scar will be scarcely noticeable. An antiseptic is needed and can best be applied in the form of a soap containing sulphur or bichloride of mercury, with which the face can be washed at night, so that the patient may avoid going into the air until the irritation caused by the antiseptic is passed away. If there is too much irritation from the use of the soap or other preparation, any of the semi-solid creams may be rubbed into the skin several times a day. Following are other good antiseptic preparations:
ity, to break up its contents. If this be done, so that the pus can

Sulphur precip. . . . . . . . . . . . . . . . . . . . . . . 1 dr.

Ether . . . . . . . . . . . . . . . . . . . . . . . . . . . . . ½ oz.

Alcohol . . . . . . . . . . . . . . . . . . . . . . . . . . . 3½ oz.

M. Sig. External use.

The lotion should at first be applied only at night, but after the skin becomes accustomed to it, it may be used advantageously several times a day. The sulphur often causes considerable irritation when first applied, but rarely so much as to cause its discontinuance.

If an ointment is desired, it may be prescribed as follows:

Sulphur precip. . . . . . . . . . . . . . . . . . . . . . . 1 dr.
Ung, aquæ rosæ,
Lanolin . . . . . . . . . . . . . . . . . . . . . . . . . . aa ½ oz.
M. Sig. External use.

Another good combination is:

Potassii sulphid.,
Zinci sulphat. . . . . . . . . . . . . . . . . . . . . . . aa 1 dr.
Aquæ . . . . . . . . . . . . . . . . . . . . . . . q. s. ad. 4 oz.
M. Sig. External use.

If the skin remains discolored after the papules and pustules have subsided, an ointment of tar and sulphur, or ichthyol and sulphur should be used, rubbing it into the skin for a half hour each night. The use of very strong stimulants, as naphthol, resorcin, caustic potash, etc., is to be avoided, as their effect is often very injurious to the skin.—*Gaillard's Medical Journal.*

